# Lactulose: an effective preventive and therapeutic option for ischemic stroke by production of hydrogen

**DOI:** 10.1186/2045-9912-2-3

**Published:** 2012-02-06

**Authors:** Xiao Chen, Xiao Zhai, Zhimin Kang, Xuejun Sun

**Affiliations:** 1 Graduates Management Unit, Changhai hospital, Second Military Medical University, Shanghai 200433, PR China; 2 Graduates Management Unit, Second Military Medical University, Shanghai 200433, PR China; 3 Department of Diving Medicine, Faculty of Naval Medicine, Second Military Medical University, Shanghai 200433, PR China

**Keywords:** lactulose, hydrogen, ischemia/reperfusion, antioxidant, stroke

## Abstract

Lactulose, a synthetic sugar not able to be digested and absorbed by human beings, is widely used to treat constipation and hepatic encephalopathy clinically. Through fermentation by the bacteria in the gastrointestinal tract, lactulose can produce considerable amount of hydrogen, which is protective for ischemic stroke as a unique antioxidant. We propose that lactulose can induce the production of endogenous hydrogen that in turn reduces oxidative stress and ameliorate the stroke damage in human beings.

## Introduction

Currently stroke is the second leading cause of death in the Western world, ranking after heart diseases and before cancer [1], causing 10% of deaths worldwide [2]. It is estimated that stroke could soon be the most common cause of death worldwide [3]. An ischemic stroke can be due to ischemia (lack of blood flow) caused by blockage (thrombosis, arterial embolism), which may lead to rapidly developing loss of brain functions as a result of disturbance in the blood supply to the brain [4]. Stroke can affect patients physically, mentally, emotionally, or a combination of the three and bring heavy burdens to society.

Ischemia induces production of reactive oxygen species (ROS), which can react with and damage a number of cellular and extracellular elements. Evidence has accumulated showing that ROS are involved in cerebral ischemia and reperfusion. During cerebral ischemia, cerebral blood flow was partially or completely cut off in brain regions supplied by the occluded vessels. Reoxygenation due to spontaneous or thrombolytic reperfusion offers oxygen as a substrate for a number of enzymatic oxidation, constantly generating ROS like superoxide anion radicals(O_2_·-) and hydrogen peroxide(H_2_O_2_) [5]. ROS are known to be able to result in macromolecular damages including lipid peroxidation, protein oxidation, and DNA oxidation, which result in ischemic brain injury [6]. Clinically, a number of recent studies have revealed that stroke and oxidative stress are closely related and excess oxidative stress may have deleterious effects on clinical outcome in acute ischemic stroke [7,8]. Therefore, antioxidants have been considered in prevention and treatment of stroke and certain agents with antioxidative effects did have neuroprotective effects [9].

### Molecular hydrogen (H_2_) serves as a novel inflammation suppressor

In recent years, experimental evidences have documented that without influencing other less potent ROS, important in intracellular signaling, molecular hydrogen possesses the ability to selectively neutralize ONOO^- ^and •OH, the most cytotoxic ROS, which can damage cellular macromolecules aggressively and indiscriminately. Thus, hydrogen can protect cells from oxidative stress injuries [10]. Therapeutic effects of hydrogen gas and hydrogen-rich saline have been experimentally confirmed in a number of studies, including hypoxia [11,12], ischemia-reperfusion injuries in various tissues and organs [13-18], and other injuries related to oxidative stress. Especially in brain ischemia, our previous research has demonstrated that hydrogen administration after hypoxia appeared to provide brain protection via inhibition of neuronal apoptosis in neonatal hypoxia-ischemia rat model [19]. Another study reported that 2.1% hydrogen-supplemented room air ventilation would preserve cerebrovascular reactivity (CR) and brain morphology after asphyxia/reventilation (A/R) in newborn pigs [20]. For transient cerebral ischemia, hydrogen also showed significant protective effects [21]. Several studies have demonstrated the neuroprotective effects of molecular hydrogen. Interestingly enough, even for some chronic neurodegenerative diseases, like the Alzheimer's disease [22] and Parkinson disease [23,24], hydrogen showed certain protective effects.

As a novel antioxidant, hydrogen possesses a number of advantages. (1) Due to its high permeability, hydrogen can easily penetrate biomembranes and diffuse into the cytosol, mitochondria and nucleus. (2) It is nontoxic to the organisms, which has been proven by hyperbaric diving study for decades. (3) Due to its selectivity as an antioxidant, hydrogen has less impact on other less active but very important ROS within the cells.

### Endogenous hydrogen is effective for alleviating oxidative stress

For therapeutic purposes, whether inhalation of hydrogen gas or injection or drinking of hydrogen-rich saline have unavoidable inconvenience. Hydrogen gas is highly flammable and explosive, thus being very dangerous. The effects of hydrogen-rich saline are not so ideally maintained. Therefore, frequent administration is required. Couldn't it be better if there is a way leading to persistent hydrogen generation under control? Endogenous hydrogen may give a perfect answer.

Early in 1969, a study published in the New England Journal of Medicine showed that endogenous hydrogen existed within human beings [25]. Studies have revealed that bacteria in the large intestine could generate endogenous hydrogen through anaerobic metabolism in human beings and animals [26]. Previously, the biologic effects of this little amount of hydrogen were often neglected, because studies have shown that the hydrogen level in normal terminal breath is about 5-10 ppm. However, in patients with lactose intolerance and bacterial disorders, the level may reach more than 90 ppm [27]. Hydrogen concentration has been measured in different organs of normal mice. The results showed that hydrogen level was very high in the large intestine, spleen, liver and gastric mucosa [28]. In the liver it reached to 42 μM, and in the large intestine and spleen it was even higher. A research on PC12 cells showed that a concentration of 25 μM of hydrogen in the medium would display a significant anti-oxidation effect [10]. These results indicated that endogenous hydrogen generated might have important biological effects.

Some of the recent findings further imply the potential therapeutic effects of endogenous hydrogen. Acarbose, which inhibits glucose absorption and often causes abdominal distention in diabetes mellitus treatment, shows an amazing cardiac protective effect. One of the main gases generated, which cause the abdominal distention, is hydrogen [29]. Oral administration of curcumin could also promote the production of endogenous hydrogen, which may be one of the mechanisms for curcumin treatment of some diseases [30]. Moreover, it is reported that oral administration of bacteria producing hydrogen gas can prevent Con A-induced hepatitis. And after antibiotic treatment, the protective effect amazingly disappeared, indicating that hydrogen produced by intestinal bacteria was the key for therapeutic effects [31].

Since hydrogen production in man is primarily dependent on the delivery of ingested, fermentable substrates to an abundant intestinal flora normally present only in the colon, identifying an ideal endogenous hydrogen inducer seems strikingly attractive.

Lactulose is a good option worth considering.

### Lactulose mediates hydrogen production and is an ideal endogenous hydrogen inducer

Lactulose is a synthetic sugar used in the treatment of constipation [32] and hepatic encephalopathy. It is a disaccharide formed from one molecule fructose and one molecule of galactose and cannot be absorbed by human bodies but can be digested by bacteria colonizing within the gastrointestinal tract, especially in the colon. One of the main byproducts is hydrogen. Oral administration of lactulose significantly increases hydrogen production [33], which can be detected by hydrogen breath test, introduced several decades ago as a diagnostic test for small bowel bacterial overgrowth [34].

In 2004, it was observed that lactulose had some protective effects on DSS-induced colitis [35]. Interestingly enough, it demonstrated that lactulose reduced the severity of colonic lesions induced by DSS treatment in a dose-dependent manner, the effect at 100 mg/kg being more potent than that of 5-ASA. Lactulose also prevented the colon shortening and ameliorated the histological inflammation, together with significant attenuation of the increase in MPO activity as well as lipid peroxidation following DSS treatment. Lactulose lowered oxidative stress state. Hydrogen may be the key.

A study showed that 20 g lactulose administration could increase the exhaled hydrogen nearly to the same level of exhaled hydrogen as compared to the consumption of 300 ml hydrogen-rich saline and had a longer maintenance time of hydrogen concentration [32]. Therefore, oral administration of lactulose may be a better option in terms of maintenance of the appropriate hydrogen gas levels in the body. Furthermore, lactulose can treat constipation by increasing the water content and volume of the stools in the bowel, making them softer and easier to pass. As is known, constipation is one of the important risk factors for cerebrovascular accidents and a major complication after stroke.

### Hypothesis

Lactulose has been proved effective in ameliorating oxidative stress injuries in DSS-induced mice colitis. Based on these observations and experiments, we hypothesize that lactulose may be a novel promising preventive and therapeutic option for stroke as an indirect antioxidant. By increasing gastrointestinal tract derived hydrogen, it may significantly reduce the possibility of stroke and alleviate ischemia/reperfusion injury after the stroke, improving the life quality of patients. What's more, it is noteworthy that lactulose probably has many other beneficial antioxidant effects on a wide range of aspects, such as cardiovascular diseases, neurodegenerative diseases et al., which still needs further study.
